# Relationships between Linear Speed and Lower-Body Power with Change-of-Direction Speed in National Collegiate Athletic Association Divisions I and II Women Soccer Athletes

**DOI:** 10.3390/sports6020030

**Published:** 2018-04-04

**Authors:** Robert G. Lockie, J. Jay Dawes, Margaret T. Jones

**Affiliations:** 1 Department of Kinesiology, California State University, Fullerton, CA 92831, USA; 2 College of Nursing and Health Sciences, University of Colorado-Colorado Springs, Colorado Springs, CO 80918, USA; jdawes@uccs.edu; 3 Health and Human Performance, George Mason University, Manassas, VA 20110, USA; mjones15@gmu.edu; 4 Center for Sports Performance, George Mason University, Fairfax, VA 22030, USA

**Keywords:** 10-m sprint, 505, agility, football, COD deficit, females, modified T-test, peak anaerobic power measured in watts, power-to-body mass ratio, vertical jump

## Abstract

This study investigated relationships that linear speed and lower-body power have on change-of-direction (COD) speed in collegiate women soccer players. Data from two Division I (*n* = 39) and one Division II (*n* = 18) schools were analyzed. Subjects were assessed in: power (vertical jump (VJ); jump height, peak anaerobic power measured in watts (PAPw), power-to-body mass ratio (P:BM); linear speed (10-m sprint); and COD speed (modified T-test (MTT), 505, COD deficit). Independent samples T-tests derived significant between-group differences, with effect sizes (*d*) calculated. Pearson’s correlations determined relationships between COD speed, linear speed, and power, with regression equations calculated. Division I players demonstrated superior 505, COD deficit, VJ height, PAPw, and P:BM (*d* = 1.09–2.21). Division II players were faster in the MTT (*d* = 1.51). For all players, the 505 correlated with the 10-m sprint (*r* = 0.39–0.53) and VJ height (*r* = −0.65–0.66), while the COD deficit related to the 10-m sprint (*r* = −0.77–0.82). The regression data supported these results. Division I players were superior in the 505 and COD deficit, and expressed their power in the 180° 505 task. Division II players should enhance lower-body power and the ability to perform 180° direction changes.

## 1. Introduction

Soccer is one of the most popular sports for women at the collegiate level [1], yet there has been relatively little analysis of these athletes [2,3,4,5,6,7,8]. Soccer places great demands on a number of physiological capacities, as players need to demonstrate high aerobic and anaerobic fitness, the ability to sprint, jump, change direction, and complete sport-specific skills such as dribbling, passing, and shooting the ball [9,10]. One of the key characteristics is the ability to change direction quickly and effectively on both offense and defense. Sheppard and Young [11] define agility as the initiation of a direction change, often in response to some form of stimulus. Change-of-direction (COD) speed is the physical component of agility, which encompasses technique, lower-body power, and the ability to effectively decelerate and accelerate [11]. This quality can help dictate performance success, due to the high volume of direction changes (e.g., more than 700 turns can be completed in a men’s professional soccer match) that are completed during soccer match-play [12]. What should also be noted is that the underlying physiological characteristics important for COD performance can differ by sex [5,13]; therefore, women athletes should be specifically analyzed.

COD speed can be task-specific [14,15], and coaches must interpret this quality within the context of the assessment utilized. Furthermore, coaches should select COD speed tests that involve movement patters inherent to the athlete’s sport. A popular test that has been used to assess COD speed is the T-test [16]. The T-test involves a forward acceleration, lateral shuffling to the left and right, and back pedaling (i.e., backwards running) [17,18,19,20]. The diversity of movement patterns within the T-test, in particular back pedaling (which does not feature in all COD speed tests) has application for soccer players [21], as they can perform these during a match [12,22]. The sprint distances within the test typically cover 4.57–9.1 m; however, Sassi et al. [18] modified the distances to 2.5–5 m in order to make it more soccer-specific. Despite its potential application, there is limited research that has analyzed the physiological factors that could impact COD speed as measured by the modified T-test (MTT) in women soccer players. McFarland et al. [5] did demonstrate that when considering the traditional T-test, greater leg power measured by the vertical jump (VJ) correlated with a faster performance in National Collegiate Athletic Association (NCAA) Division II women soccer players (correlation coefficient (*r*) = −0.76). Nonetheless, further research is required to assess the physiological capacities that could influence performance in a test featuring linear acceleration, lateral shuffling, and back pedaling, as these movement patterns feature prominently in soccer [10].

Another test commonly used to assess COD speed is the 505 [23,24]. The 505 generally involves a 10-m sprint past a timing gate, a further 5-m sprint to a turning line where the athlete completes a 180° cut, before sprinting back through the gate [24]. The 505 can isolate COD ability for each leg [17,25,26], and has been used accordingly to assess this capacity in soccer players [27,28,29]. Again, this test has application as a soccer player can be required to perform this type of direction change during a match [12]. However, the underlying physiological characteristics important to performing a 180° cut have not been clearly determined. Jones et al. [30] linked greater eccentric knee extensor strength to a faster 180° cut in elite women soccer players, but did not assess linear speed and lower-body power. In softball players, Nimphius et al. [25] found that linear speed over 10 m and 17.9 m correlated with the 505 performed from the non-dominant leg (*r* = 0.76 and 0.86, respectively), but not the dominant leg (*r* = 0.55 and 0.66, respectively). Nimphius et al. [25] also documented that VJ performance did not correlate with the 505 times (*r* = −0.25 to −0.35). Further research is required to document whether or not linear speed and lower-body power influences performance on the 505 test in NCAA women soccer players.

The 505 can be used to produce another measure of COD ability, which has been termed the COD deficit [23]. Nimphius et al. [23] defined the COD deficit as the difference between the average 505 time and 10-m sprint time. This measure calculates the impact that implementing a 180° cut has on the ability to cover a 10-m distance, with a smaller COD deficit indicating a better ability to change direction. The COD deficit may be able to provide an assessment of COD ability independent of linear speed [14,23,24,31], although this requires further investigation. In men cricketers, the COD deficit did not correlate to sprint performance over 10 m and 30 m for either the preferred (*r* = −0.10 and −0.11, respectively) or non-preferred (*r* = −0.08 and −0.10, respectively) leg. Lockie et al. [29] found that the COD deficit for either the left (*r* = −0.21 to 0.38) or right (*r* = −0.22 to 0.07) leg did not correlate with either the VJ, triple hop, or standing broad jump in NCAA Division I collegiate men soccer players. The COD deficit may exhibit different relationships to linear speed and lower-body power in collegiate women soccer players.

A further consideration for collegiate women soccer players is that COD speed, and the effects that linear speed and lower-body power may have on this quality, could vary between players across different divisions of play. The highest level of NCAA competition is Division I, followed by Divisions II and III. It could be expected that athletes from Division I may exhibit superior COD ability, linear speed and lower-body power, as this has been shown in collegiate football players [32,33]. However, soccer is a non-revenue generating sport for most schools, and soccer players may select schools for reasons other than just athletics [34]. This could result in women soccer athletes from different NCAA levels of player to be more similar in certain physiological characteristics, although no research has investigated this. A greater understanding of COD speed in Division I and II women soccer players, and the potential influence that linear speed and lower-body power can have on these qualities, could help inform the strength and conditioning practices adopted by the coaches of collegiate teams from all levels of NCAA play.

Therefore, this study investigated how linear speed and lower-body power may influence COD speed, as measured by the MTT, 505, and COD deficit, in NCAA Divisions I and II women soccer players. It was hypothesized that Division I athletes would be faster in the MTT, 505, 10-m sprint, COD deficit, and would demonstrate a superior VJ performance and greater power. It was further hypothesized that greater linear speed and power measured from the VJ would correlate with faster MTT, 505, and COD deficit performance in both Divisions I and II women soccer players.

## 2. Materials and Methods

### 2.1. Subjects

The coaching and support staff collected data from two Division I and one Division II soccer team. This sample of convenience comprised 57 women (age: 19.9 ± 1.3 years; height: 1.65 ± 0.62 m; body mass: 63.7 ± 7.3 kg). The subjects were required to be: a member of the respective school’s playing squad and a field player (i.e., defender, midfielder, or forward); over 18 years of age; injury-free; and currently completing full training at the time of testing (~17 h per week) [3]. Goalkeepers were excluded from this analysis due to dissimilar movement patterns when compared to field players [12]. G*Power software (v3.1.9.2, Universität Kiel, Düsseldorf, Germany) confirmed that a sample size of 57 was satisfactory for an independent samples T-test analysis. This process ensured the data could be interpreted with a power level of 0.70 when significance was set at 0.05 [35], and a moderate effect level of 0.58 [36]. Furthermore, G*Power software was also used to confirm that for a correlation, point biserial model, sample sizes of 39 and 18 ensured the data could be interpreted with small (0.37) and moderate (0.53) effect levels, respectively [36], when the power level was 0.81 and significance was set at 0.05 [35]. Subjects were medically cleared for intercollegiate athletic participation at their respective academic institution, and read and signed the university consent and medical forms for participation in collegiate athletics. One of the institutions that provided subjects for this study had ethics approval for data collection. These subjects had the risks and benefits explained to them beforehand, and informed consent was obtained. The university’s institutional ethics committee approved all procedures. For the two other schools that provided subjects for this research, data were analyzed retrospectively. Based upon the archival nature of these analyses, the institutional ethics committee for these schools approved the use of pre-existing data. Nevertheless, all aspects of this study conformed to the recommendations of the Declaration of Helsinki.

### 2.2. Procedures

Data for all teams were collected during the non-traditional period (i.e., the off-season where players practice but do not play matches) of the collegiate soccer season, and the subjects from all schools were familiar with the tests. Only those data that were collected with the same methods were incorporated into this study. The procedures for the tests conducted in this study have been published previously [2,3,4,5,28,29]. For each team, age, height, and body mass were recorded prior to testing. All tests were preceded by a dynamic warm-up, which generally consisted of 10 min of low-intensity activity, and 10 min of dynamic stretching of the lower limbs, and linear and lateral runs over approximately 20 m that progressively increased in intensity. For all schools, the VJ was performed indoors on a firm surface. Speed testing was conducted either indoors, or on field turf or natural grass surfaces. Subjects wore their own shoes for all tests, which were either athletic trainers or team-issued soccer flats for the VJ, or team-issued soccer flats or cleats for the speed tests. Subjects were permitted to consume water as necessary during the testing session.

### 2.3. Vertical Jump (VJ)

The VJ was measured at all schools via a jump mat (Just Jump, Probotics Inc., Huntsville, AL, USA) [3,5,28]. The protocols used for all schools have been shown to have high reliability, as documented by intra-class correlation coefficients (ICCs) greater than 0.9, and coefficients of variation (CVs) less than 5% [37]. The subject initially stood on the jump mat keeping her heels on the mat, before completing a countermovement and jumping as high as possible. No restrictions were placed on the countermovement range of motion. Subjects were free to swing their arms during the jump, and were instructed to maintain straight legs during the flight, before landing on both feet with flexion of the hips, knees, and ankles. Within the software for the mat, jump height in centimeters (cm) was calculated from flight time via the following equation: *jump height* = (½ × *acceleration due to gravity* (−9.81 m·s^2^) × (*total flight time* ÷ 2)^2^). Each subject completed 2–3 trials, and the best trial was used for analysis. The peak anaerobic power measured in watts (PAPw) from the VJ was also calculated for the best trial by using the Sayers et al. [38] equation: PAPw = (60.7 VJ *height* (cm)) + (45.3·*body mass* (kg)) − 2055. PAPw was also calculated relative to body mass to provide a power-to-body mass ratio (P:BM) via the equation: P:BM = PAPw·BM^−1^ [5,39].

### 2.4. Ten-Meter Sprint

The 10-m sprint was used to measure sprint acceleration. This was the only measured sprint interval that was consistent across the three schools. Additionally, 10-m sprint performance has been used to measure the acceleration performance of collegiate soccer players in a number of studies [2,3,4,5,28,29,40]. Time was recorded by a timing lights system (Fusion Sports, Sumner Park, Australia or TC Timing System, Brower Timing, Draper, UT, USA). Although it is not ideal to compare the times recorded by two different systems, both systems have been found to be reliable and valid [41,42,43,44,45]. Indeed, the protocols used in this research have been found to produce ICCs of ≥0.9 and CVs of ≤3% for the Fusion Sports [44,45] and Brower systems [41,42]. Further to this, previous research has noted the linear sprint speed over 36.6 m performed by collegiate male football players was not significantly different when performed on field turf or grass [46]. Timing gates positioned at 0 m and 10 m to record 10-m sprint time. Subjects began the sprint from a standing start 50 cm behind the start line to trigger the first gate, and were instructed to run maximally after they initiated their sprint. 2–3 trials were completed, time for each interval was recorded to the nearest 0.01 s, and the fastest trial was used for analysis.

### 2.5. Modified T-Test (MTT)

**Figure 1:**
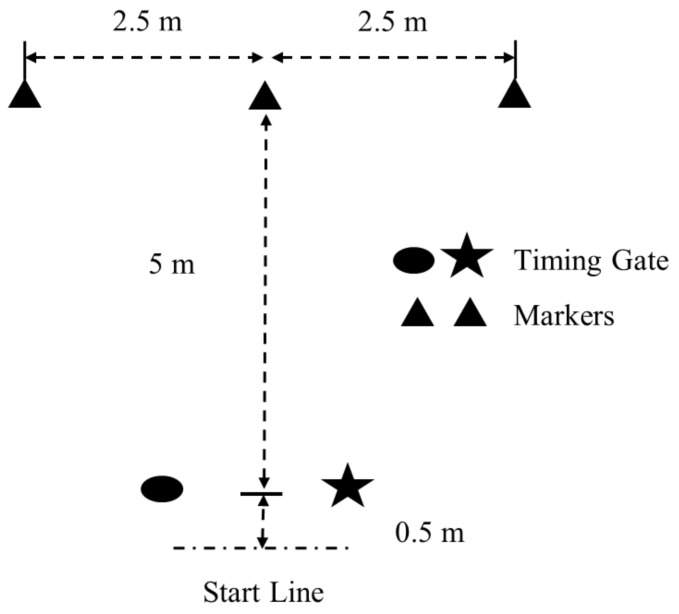
Structure of the modified T-test.

The MTT was used in this study [18,19], and is shown in Figure 1. The MTT was utilized as a COD assessment in this study because it incorporates soccer-specific movements such as sprint accelerations, decelerations, lateral shuffling, and back pedaling [10,17,18,19,20]. The procedures utilized in this study have yielded an ICC of 0.92 and CV of 2.6% in college-aged women [18]. One timing gate was utilized (Fusion Sports, Sumner Park, Australia or TC Timing System, Brower Timing, Draper, UT, USA), the subjects for all schools started 50 cm behind the start line, and faced forwards at all times during the test. As described by Lockie et al. [19], subjects sprinted forwards through the gate to touch the middle marker, side-shuffled 2.5 m to the left or right, depending on the trial, to touch the next marker, side-shuffled 5 m in the other direction to touch the next marker, side-shuffled 2.5 m back to touch the middle marker, before back-pedaling past the timing gate to finish. The hand that was on the same side as the shuffle direction (i.e., the right hand when shuffling to the right, and the left hand when shuffling to the left) was used to touch the marker. Subjects were not to cross their feet when side-shuffling; if they did, the trial was stopped and reattempted. Few trials across all teams needed to be reattempted, but this was always done after a sufficient recovery period (i.e., greater than 3 min). Subjects completed 2–3 trials initiating movement to the left or right, for 4–6 trials total. The order of which direction movement was initiated in the MTT (i.e., left or right) trials was randomized. Time for was measured to the nearest 0.01 s, and the fastest trial was used in this study.

### 2.6. The 505 Test

**Figure 2:**
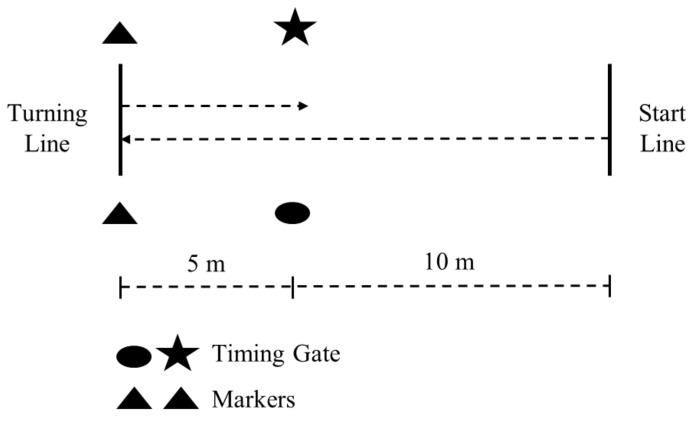
Structure of the 505 test.

The methodology for the 505 was conducted per established methods for each school [23,24,25,26,28,29]. The dimensions for the 505 are shown in Figure 2. Subjects used a standing start with the same body position as for the 10-m sprint. The subjects sprinted through the timing gate (Fusion Sports, Sumner Park, Australia or TC Timing System, Brower Timing, Draper, UT, USA) to the turning line. Subjects placed either the left or right foot, depending on the trial, on or behind the turning line, before sprinting back through the gate. 2–3 trials were recorded for turns off the left and right leg (4–6 trials total), the order of which was randomized. A coach was positioned at the turning line, and if the subject changed direction before hitting the turning point, or turned off of the incorrect foot, the trial was disregarded and reattempted. Time for each trial was recorded to the nearest 0.01 s, and the fastest trial was used for analysis. COD deficit for each leg was calculated via the formula: 505 time–10-m time [23]. The 10-m time was taken from the linear speed test.

### 2.7. Statistical Analysis

All statistical analyses were computed using the Statistics Package for Social Sciences (Version 24.0; IBM Corporation, New York, NY, USA), and stem-and-leaf plots confirmed a normal data distribution for each variable. Descriptive statistics (mean ± standard deviation (SD) were calculated for each test parameter. As stated, subjects were stratified into Division I or Division II groups to allow for comparisons between the performance tests. Independent samples T-tests were used to compute any significant differences between the groups, and an alpha level of *p* < 0.05 was required for significance. Effect sizes (*d*) were also calculated for the between-group comparisons, where the difference between the means was divided by the pooled SD [47]. In accordance with Hopkins [36], a *d* less than 0.2 was considered a trivial effect; 0.2–0.6 a small effect; 0.6–1.2 a moderate effect; 1.2–2.0 a large effect; 2.0–4.0 a very large effect; and 4.0 and above an extremely large effect.

The relationships between the linear speed (10-m sprint) and jump tests (VJ, PAPw, and P: BM) with the COD speed tests (MTT, 505, and COD deficit) was also investigated via Pearson’s two-tailed correlation analysis. Divisions I and II players were analyzed separately, and an alpha level of *p* < 0.05 was again required for significance. The correlation strength was designated as per Hopkins [48]: an *r* between 0 to 0.3, or 0 to −0.3, was considered small; 0.31 to 0.49, or −0.31 to −0.49, moderate; 0.5 to 0.69, or −0.5 to −0.69, large; 0.7 to 0.89, or −0.7 to −0.89, very large; and 0.9 to 1, or −0.9 to −1, near perfect for relationship prediction. Furthermore, multiple regressions were calculated for the pooled data, and Divisions I and II players separately, to predict each COD speed measurement (i.e., MTT, 505, and COD deficit) based on VJ, PAPw, P:BM, and 10-m sprint performance.

## 3. Results

The data for all assessments are displayed in Table 1. The NCAA Division I players were significantly older than the Division II players (moderate effect). No significant differences in height or body mass were discovered, both of which had trivial effects. The Division I players had significantly greater VJ scores (moderate), generated greater PAPw (moderate), and had a superior P:BM ratio (very large) when compared to the Division II players. No significant differences were found in 0–10 m time between groups. In regard to COD speed, Division I players performed significantly better on the 505, and displayed a lower COD Deficit (both moderate) when compared to DII players. Interestingly, while the Division I players were superior in the 505, Division II players were significantly faster on the MTT, which had a large effect.

The correlation data for Division I and II groups are shown in Table 2 and Table 3, respectively. In the Division I players, the MTT did not significantly relate to the 10-m sprint or any of the VJ variables. The 505 positively correlated with the 10-m sprint (moderate), and negatively correlated with VJ height and P:BM (both large). COD deficit very large negative relationship with the 10-m sprint. For the Division II players, the MTT had large relationships with the 10-m sprint, VJ height, and P:BM. The 505 had large relationships with the 10-m sprint and all the VJ variables, with COD deficit correlated very largely with the 10-m sprint.

Results of the multiple linear regression model using the pooled sample revealed a significant regression equation (F (1, 55) = 65.659, *p* < 0.001), with an R^2^ of 0.55 for 505 and VJ. A significant regression equation was also found for the COD deficit and 10-m sprint and VJ (F (1, 55) = 80.568, *p* < 0.001), with an R^2^ of 0.75. No significant regression equation for the MTT and any of the other measured variables were discovered for the pooled data.

When each division level was analyzed separately, it was discovered that Division I players both VJ and PAPw (F (1, 37) = 26.876, *p* < 0.001), explained 46% of the variance in 505 Test performances. Additionally, it was found that the 79% of the variance in the COD deficit was explained by the player’s 10-m sprint time and P:BM (F (1, 37) = 66.761, *p* < 0.001). Among Division II players, it was found that 43% of the variance in MTT test performance was explained by 10-m sprint time (F (1, 16) = 11.834, *p* = 0.003), and 44% of the variance in 505 test performance could be explained by VJ (F (1, 16) = 12.346, *p* = 0.003). Furthermore, it was also discovered that 10-m sprint time and PAPw (F (2, 15) = 20.279, *p* < 0.001) accounted for 69% of the COD deficit time.

## 4. Discussion

The current study investigated how linear speed and leg power may influence COD speed in NCAA Divisions I and II women soccer players. There has been little analysis of collegiate athletes across different levels of play [32,33], and no analysis of women soccer players. The Division I players from the current study were older than the Division II players, however, all were within the typical range of collegiate soccer players documented in the literature [3,5,28,29,40,49]. The Division I players from this study were faster in the 505 and had a lower COD deficit, in conjunction with a superior VJ, PAPw, and P:BM. Conversely, the Division II players were faster in the MTT, and linear speed and lower-body power correlated with this variable for these players. Strength and conditioning coaches should be cognizant that the extent lower-body power could influence COD performance in collegiate women soccer players may be dependent on the type of direction change required within a task.

Division I players were faster in the 505, and also demonstrated greater lower-body power as measured by VJ height, PAPw, and P:BM. Furthermore, faster 505 related to a faster 10-m sprint and VJ height in both Divisions I and II players, and the 505 was predicted by the VJ height and PAPw for the pooled sample and Division I players, and VJ height for Division II players. Lower-body power and reactive strength are said to be part of the foundation of COD speed [11]. Further, the up-and-back COD (i.e., 180° cut) that is required in the 505 can be quite challenging to an athlete, especially when compared to direction changes of lesser angles [14]. A faster athlete in a task such as the 505 will need to have superior neuromuscular qualities. Indeed, Delextrat and Cohen [50] documented that women basketball players who were faster in a run featuring multiple 180° direction changes exhibited higher power as measured by a single-leg jump. Further, Spiteri et al. [51] found eccentric strength was a key contributor to faster 505 performance in a similar population. Specific to soccer players, Jones et al. [30] suggested that greater eccentric knee extensor strength contributed to a faster 180° cut in elite women. It is likely that the Division I players had greater neuromuscular qualities as measured by the VJ power, and this provided a positive influence on 505 test performance, which was supported by the correlation and regression data.

Nimphius et al. [23] proposed that the COD deficit provided an alternative measure of COD ability, independent of linear speed. Interestingly, for both the Divisions I and II players, there was a negative relationship between COD deficit and the 10-m sprint. This could relate to the fact that 10-m sprint time was used to calculate COD deficit [23]. Indeed, the COD deficit was predicted by 10-m sprint time for the total subject sample, and Divisions I and II players when analyzed separately, which highlights the contribution of this metric to the COD deficit. Nonetheless and more notably, the results indicated that the Division I players had a significantly lower COD deficit when compared to the Division II players, which meant the impact that the 180° direction change had on their ability to cover the 10-m distance in the 505 was less than that for the Division II players. The ability to execute an effective direction change requires eccentric strength in order to decelerate the body, followed by concentric force development for reacceleration in the new intended direction [52,53]. The Division I women soccer players were likely able to perform these actions more effectively than their Division II counterparts, which led to the lower COD deficit. As for the 505, the neuromuscular qualities as measured by VJ height, PAPw, and P:BM would also have positively contributed to the ability to change direction more quickly, given the importance of power to COD speed [11]. This was also reinforced by the regression data, where the COD deficit was also predicted by the VJ for all players combined, and P:BM in Division I players, and PAPw in Division II players. As previously discussed, these predictive relationships highlight the contributions that lower-body power and eccentric strength, as measured by VJ height, PAPw, and P:BM, have on the effectiveness of a direction change [30,50,51,52,53]. Greater superiority in these qualities could have allowed the subject to more effectively load the leg when executing the direction change [14], and subsequently more quickly and explosively navigate the 180° cut in the 505.

Despite demonstrating lesser lower-body power when compared to the Division I group, the Division II players were faster in the MTT. A contributing factor could be that the MTT for the Division II players was completed on field turf, as opposed to grass for the Division I players. Division II male college football players were found to complete the pro-agility shuttle significantly faster on field turf when compared to natural grass [46]. However, the effect for the difference documented by Gains et al. [46] was small (*d* = ~0.38); the difference in the MTT between the Divisions I and II groups in this research was large (*d* = 1.51). This would suggest that there was still a meaningful different in MTT performance between the Divisions I and II groups, regardless of the testing surface. Further to this, there were no significant correlations between linear speed and lower-body power for the Division I players. For the Division II players, a faster MTT related to a faster 10-m sprint, and greater VJ height and P:BM. MTT was also predicted by the 10-m sprint in Division II, but not the Division I, players. Nimphius et al. [14] discussed how the direction of change required in a COD task can change the inherent demands of the test. The direction changes required in the MTT were approximately 90°, as opposed to the 180° COD featured in the 505. Potentially, the Division II players were able to express their linear speed and lower-body power within the task demands of the MTT. However, these players may not have been able to do this relative to the more difficult COD task required in the 505 [14].

There are study limitations that should be acknowledged. This study incorporated teams from three universities, so the results may only be representative of athletes from these schools. Furthermore, the testing conditions, number of trials, and surfaces differed slightly, which is not uncommon when using comparative data between Divisions I and II institutions [32,33]. Only two COD speed tests was analyzed in this study; it would be of interest to investigate other COD speed tests used to assess this capacity in collegiate women’s soccer players (e.g., pro-agility shuttle, Arrowhead) [2,3,4,6,7,54]. Strength was not measured in this study, and this could potentially be a differentiating factor between Divisions I and II women soccer players [32,33], and could have some influence on COD speed [52,53]. Future research should measure the lower-body strength of women soccer players, either via repetition-maximum tests or the isometric mid-thigh pull, to ascertain any possible effects on COD speed. It would also be valuable to conduct the analysis from this study at different time points within a collegiate season, to illustrate whether any of the relationships between COD speed, linear speed, and lower-body power changes during different phases of the training cycle. Nevertheless, within the context of these limitations, this study found that Division I collegiate women soccer players demonstrated superior COD speed as measured by the 505 and COD deficit, and greater lower-body power as measured by VJ height, PAPw, and P:BM. Although Division II players were superior in the MTT and linear speed and VJ height correlated with this test, Division II players should improve their lower-body power and COD ability so can navigate difficult 180° direction changes more effectively.

## 5. Conclusions

In conclusion, Division II women soccer players may display lesser lower-body power as measured by the VJ when compared to Division I players. The results from this study suggested that this could influence their ability to change direction quickly in difficult 180° turns. This should be a point of emphasis for strength and conditioning coaches of players from this level of NCAA competition. Division II women soccer players should attempt to enhance their lower-body power such that it could positively influence COD speed, and the ability to complete difficult 180° direction changes. Accordingly, coaches for Division II women soccer players should ensure that their players have the requisite neuromuscular qualities in order to quickly and effectively navigate more difficult COD tasks which can be required during match-play. Training protocols including specific COD drills and plyometrics could assist with this process [55,56,57]. Division I women soccer players had faster COD speed as define by the 505 and COD deficit. As the COD deficit is a recent introduction as an assessment tool for COD ability [14,23], this should be analyzed further in women soccer players. Given the volume of direction changes completed in soccer matches at all levels of play [12], it is essential that metrics such as the COD deficit are investigated with regards to their utility in this population, including collegiate women players. However, it should be acknowledged that the Division I players in the current study were slower in the MTT when compared to the Division II players. It is recommended that NCAA Division I strength and conditioning coaches ensure their soccer athletes can sustain COD speed over relatively longer durations of ~6 s.

## Figures and Tables

**Table 1:** Descriptive data (mean ± SD) for NCAA Division I and Division II women soccer players for the vertical jump (VJ), VJ peak anaerobic power measured in watts (PAPw), PAPw-to-body mass ratio (P:BM), and times for the 0–10 m sprint, modified T-test (MTT), 505, and COD deficit.

Variables	Division I (*n* = 39)	Division II (*n* = 18)	*p*	*d*	*d* Strength
Age (years)	20.19 ± 1.19	19.33 ± 1.23	0.16	0.71	Moderate
Height (m)	1.64 ± 0.07	1.65 ± 0.05	1.00	0.16	Trivial
Body Mass (kg)	63.83 ± 7.81	63.31 ± 6.25	1.00	0.07	Trivial
VJ (cm)	47.19 ± 6.69	39.99 ± 5.39 *	<0.01	1.19	Moderate
PAPw (w)	3701.03 ± 428.10	3240.30 ± 418.56 *	0.01	1.09	Moderate
P:BM (w·kg^−1^)	66.00 ± 7.72	51.26 ± 5.39 *	<0.01	2.21	Very Large
0–10 m (s)	1.91 ± 0.16	1.89 ± 0.17	1.00	0.12	Trivial
MTT (s)	6.93 ± 0.44	6.25 ± 0.46 *	<0.01	1.51	Large
505 (s)	2.40 ± 0.10	2.60 ± 0.11 *	<0.01	1.90	Moderate
COD Deficit (s)	0.49 ± 0.16	0.72 ± 0.18 *	<0.01	1.35	Moderate

* Significantly (*p* < 0.05) different from the Division I group.

**Table 2:** Correlations between the 10-m sprint (linear speed), vertical jump (VJ) height, peak anaerobic power measured in watts (PAPw), and PAPw-to-body mass ratio (P:BM), with the modified T-test, 505, and COD deficit in NCAA Division I women soccer athletes (*n* = 39).

Variables	10-m Sprint	VJ Height	PAPw	P:BM
Modified T-test	−0.33	0.18	−0.43	0.22
505 Test	0.39 *	−0.65 **	−0.64 **	−0.65 **
COD Deficit	−0.82 **	−0.01	0.05	−0.04

* Significant (*p* < 0.05) relationship between the two variables. ** Significant (*p* < 0.01) relationship between the two variables.

**Table 3:** Correlations between the 10-m sprint (linear speed), vertical jump (VJ) height, peak anaerobic power measured in watts (PAPw), and PAPw-to-body mass ratio (P:BM), with the modified T-test, 505, and COD deficit in NCAA Division II women soccer athletes (*n* = 18).

Variables	10-m Sprint	VJ Height	PAPw	P:BM
Modified T-test	0.65 *	−0.52 *	−0.29	−0.52 *
505 Test	0.55 *	−0.66 **	−0.64 **	−0.63 *
COD Deficit	−0.77 **	0.07	−0.09	0.11

* Significant (*p* < 0.05) relationship between the two variables. ** Significant (*p* < 0.01) relationship between the two variables.

## Abbreviations

The following abbreviations are used in this manuscript:

COD	Change-of-direction
m	Meter
MTT	Modified T-test
VJ	Vertical jump
NCAA	National Collegiate Athletic Association
*r*	Correlation coefficient
kg	kilograms
ICC	Intra-class correlation coefficient
CV	coefficient of variation
cm	Centimeters
PAPw	Peak anaerobic power measured in watts
P:BM	Power-to-body mass ratio
s	Second
SD	Standard deviation
*p*	Significance
*d*	Effect size
w	watts
w·kg^−1^	watts per kilogram body mass
